# Antibiotic Prescribing in Acute Wound Management

**DOI:** 10.1192/bjo.2024.558

**Published:** 2024-08-01

**Authors:** Hani Dourado Al-khatib, Emma Corn, Victoria Ozidu

**Affiliations:** Greater Manchester Mental Health, Manchester, United Kingdom

## Abstract

**Aims:**

The primary aim is to ensure patients receive recommended acute wound care. Specific objectives include improving wound management, enhancing antimicrobial stewardship, and aligning practices with national guidelines.

**Methods:**

A retrospective audit spanning May to October 2023 assessed prescriptions for in-patients receiving antibiotics for wound management. Detailed patient records were scrutinized to evaluate compliance with standards, including wound assessment documentation, antibiotic indication adherence, tetanus status recording, and wound swab collection.

**Results:**

A total of 21 patients/encounters met the criteria for inclusion. Documentation deficiencies were prevalent, with only 61.9% of prescriptions featuring complete wound assessments. Additionally, antibiotic indications met NICE criteria in only 42.8% of cases, while tetanus status documentation was absent across all records. Despite 76% receiving first-line antibiotics, only 19% had wound swabs collected.

**Conclusion:**

Self-harm rates in the United Kingdom, particularly among those with mental health disorders, are alarming. Hospitalizations are often required to address acute self-inflicted wounds, yet in-patient settings present unique challenges exacerbating self-harming tendencies.

This audit underscores the imperative of optimizing acute wound management in in-patient settings. By implementing evidence-based practices and addressing identified deficiencies, healthcare providers can enhance patient outcomes and ensure optimal care delivery.

